# Supplementary material for: Assessing population structure and morpho-molecular characterization of sunflower (Helianthus annuus L.) for elite germplasm identification
Source: PeerJ. 2024 Oct 31;12:e18205. doi: 10.7717/peerj.18205 (PMC11531741; doi:10.7717/peerj.18205)
Supplement: Table S1 [file peerj-12-18205-s003.docx]

Supplementary Table 1: The list of 48 sunflower germplasms and their sources used in the genetic diversity study.

| **S.NO** | **Germplasms** | **Source** |
| --- | --- | --- |
| 1 | ARM 248B | IIOR, Hyderabad |
| 2 | COSF 6B © | TNAU, Coimbatore |
| 3 | GMU 1181 | IIOR, Hyderabad |
| 4 | RHA 102 | UAS, Bangalore |
| 5 | *CMS* 1103B | UAS, Bangalore |
| 6 | GMU 780 | IIOR, Hyderabad |
| 7 | CSFI 99 © | TNAU, Coimbatore |
| 8 | *CMS* 335B | UAS, Bangalore |
| 9 | RCR 72 | UAS, Raichur |
| 10 | *CMS*ND*CMS*2B | RARS, Nandyal |
| 11 | GMU 344 | IIOR, Hyderabad |
| 12 | RHA 272-1 | UAS, Bangalore |
| 13 | GMU 336 | IIOR, Hyderabad |
| 14 | RHA GPR 58 | UAS, Bangalore |
| 15 | COSFV 5 | TNAU, Coimbatore |
| 16 | *CMS* 911B | UAS, Bangalore |
| 17 | GMU 477 | IIOR, Hyderabad |
| 18 | GMU 450 | IIOR, Hyderabad |
| 19 | PM 95 | UAS, Raichur |
| 20 | GMU 428 | IIOR, Hyderabad |
| 21 | GMU 411 | IIOR, Hyderabad |
| 22 | GMU 741 | IIOR, Hyderabad |
| 23 | *CMS* 108B | UAS, Bangalore |
| 24 | GMU 734 | IIOR, Hyderabad |
| 25 | ARM 240B | IIOR, Hyderabad |
| 26 | *CMS* 597B | UAS, Bangalore |
| 27 | HOCL 6R | UAS, Bangalore |
| 28 | RHA GPR 110 | UAS, Bangalore |
| 29 | IB 80 | PAU, Ludhiana |
| 30 | *CMS* 107B | UAS, Bangalore |
| 31 | PM 36 | UAS, Raichur |
| 32 | IL 77 | PAU, Ludhiana |
| 33 | RHA95-C-10 | UAS, Bangalore |
| 34 | IL84 | PAU, Ludhiana |
| 35 | RHA 278 | UAS, Bangalore |
| 36 | RHA 273 | UAS, Bangalore |
| 37 | RHA 857 | UAS, Bangalore |
| 38 | GMU 755 | IIOR, Hyderabad |
| 39 | PM 53 | UAS, Raichur |
| 40 | GP6 912 | IIOR, Hyderabad |
| 41 | *CMS* 135B | UAS, Bangalore |
| 42 | REC 431 | IIOR, Hyderabad |
| 43 | GMU 325 | IIOR, Hyderabad |
| 44 | GP6 1089 | IIOR, Hyderabad |
| 45 | RHA 378 | UAS, Bangalore |
| 46 | PM 65 | UAS, Raichur |
| 47 | RHA GMU 755 | IIOR, Hyderabad |
| 48 | COSF 13B | TNAU, Coimbatore |

IIOR: Indian institute of oil seed research; TNAU: Tamil Nadu agricultural university; UAS: University of agricultural science; PAU: Punjab agricultural university and RARS: Regional agricultural research station
