# Supplementary material for: Assessing population structure and morpho-molecular characterization of sunflower (Helianthus annuus L.) for elite germplasm identification
Source: PeerJ. 2024 Oct 31;12:e18205. doi: 10.7717/peerj.18205 (PMC11531741; doi:10.7717/peerj.18205)
Supplement: Table S2 [file peerj-12-18205-s004.docx]

Supplementary Table 2: Genotyping data using SSR markers across the studied sunflower genotyes.

| S.no | Primers | | band size | TB | PB | % P | AvgPIC | PI | (Rp) | MEAN Rp |
| --- | --- | --- | --- | --- | --- | --- | --- | --- | --- | --- |
| 1 | ORS8 | | 250-450 | 2 | 2 | 100 | 0.489 | 0.978 | 2.292 | 1.146 |
| 2 | ORS148 |  | 200-250 | 2 | 2 | 100 | 0.395 | 0.790 | 2.000 | 1.000 |
| 3 | ORS185 |  | 300-420 | 4 | 4 | 100 | 0.375 | 1.498 | 2.333 | 0.583 |
| 4 | ORS200 |  | 310-520 | 2 | 2 | 100 | 0.265 | 0.530 | 1.542 | 0.771 |
| 5 | ORS203 |  | 200-220 | 2 | 2 | 100 | 0.388 | 0.777 | 1.292 | 0.646 |
| 6 | ORS244 |  | 160-420 | 4 | 4 | 100 | 0.244 | 0.975 | 3.375 | 0.844 |
| 7 | ORS307 |  | 160-180 | 2 | 2 | 100 | 0.432 | 0.864 | 1.292 | 0.646 |
| 8 | ORS309 |  | 140-220 | 3 | 3 | 100 | 0.345 | 1.036 | 2.000 | 0.667 |
| 9 | ORS310 |  | 180-220 | 3 | 3 | 100 | 0.400 | 1.201 | 2.500 | 0.833 |
| 10 | ORS317 |  | 200-240 | 2 | 2 | 100 | 0.201 | 0.402 | 1.875 | 0.938 |
| 11 | ORS321 |  | 180-920 | 3 | 3 | 100 | 0.306 | 0.919 | 2.458 | 0.819 |
| 12 | ORS328 |  | 320-340 | 2 | 2 | 100 | 0.253 | 0.506 | 0.625 | 0.313 |
| 13 | ORS323 |  | 390-420 | 2 | 2 | 100 | 0.165 | 0.330 | 2.417 | 1.208 |
| 14 | ORS331 |  | 320-420 | 3 | 3 | 100 | 0.397 | 1.191 | 2.250 | 0.750 |
| 15 | ORS333 |  | 200-370 | 3 | 3 | 100 | 0.243 | 0.728 | 2.792 | 0.931 |
| 16 | ORS334 |  | 310-330 | 2 | 2 | 100 | 0.153 | 0.306 | 2.000 | 1.000 |
| 17 | ORS337 |  | 200-210 | 2 | 2 | 100 | 0.249 | 0.498 | 2.000 | 1.000 |
| 18 | ORS342 |  | 210-230 | 2 | 2 | 100 | 0.114 | 0.227 | 1.833 | 0.917 |
| 19 | ORS351 |  | 290-300 | 2 | 2 | 100 | 0.345 | 0.691 | 1.750 | 0.875 |
| 20 | ORS358 |  | 120-328 | 3 | 3 | 100 | 0.394 | 1.182 | 3.000 | 1.000 |
| 21 | ORS366 |  | 180-190 | 2 | 2 | 100 | 0.408 | 0.816 | 2.750 | 1.375 |
| 22 | ORS378 |  | 200-240 | 2 | 2 | 100 | 0.412 | 0.825 | 1.167 | 0.583 |
| 23 | ORS384 |  | 280-400 | 4 | 4 | 100 | 0.368 | 1.474 | 2.917 | 0.729 |
| 24 | ORS386 |  | 220-320 | 2 | 2 | 100 | 0.164 | 0.329 | 2.208 | 1.104 |
| 25 | ORS425 |  | 1020 | 2 | 2 | 100 | 0.430 | 0.430 | 0.625 | 0.625 |
| 26 | ORS426 |  | 160-200 | 2 | 2 | 100 | 0.316 | 0.631 | 2.458 | 1.229 |
| 27 | ORS437 |  | 320 | 2 | 2 | 100 | 0.457 | 0.457 | 1.292 | 1.292 |
| 28 | ORS488 |  | 220-420 | 2 | 2 | 100 | 0.201 | 0.402 | 1.875 | 0.938 |
| 29 | ORS502 |  | 160-200 | 3 | 3 | 100 | 0.315 | 0.944 | 2.792 | 0.931 |
| 30 | ORS511 |  | 160-200 | 3 | 3 | 100 | 0.117 | 0.350 | 2.042 | 0.681 |
| 31 | ORS543 |  | 220-230 | 2 | 2 | 100 | 0.401 | 0.801 | 1.458 | 0.729 |
| 32 | ORS547 |  | 180-190 | 2 | 2 | 100 | 0.299 | 0.597 | 1.500 | 0.750 |
| 33 | ORS552 |  | 160-200 | 3 | 3 | 100 | 0.315 | 0.944 | 3.708 | 1.236 |
| 34 | ORS558 |  | 280-290 | 2 | 2 | 100 | 0.338 | 0.676 | 1.542 | 0.771 |
| 35 | ORS559 |  | 200-550 | 3 | 3 | 100 | 0.217 | 0.651 | 0.917 | 0.306 |
| 36 | ORS565 |  | 160-180 | 2 | 2 | 100 | 0.201 | 0.402 | 3.542 | 1.771 |
| 37 | ORS578 |  | 200-520 | 2 | 2 | 100 | 0.234 | 0.468 | 1.958 | 0.979 |
| 38 | ORS581 |  | 280-620 | 4 | 4 | 100 | 0.422 | 1.687 | 3.458 | 0.865 |
| 39 | ORS591 |  | 380-900 | 4 | 4 | 100 | 0.356 | 1.781 | 4.292 | 0.858 |
| 40 | ORS595 |  | 120-140 | 2 | 2 | 100 | 0.344 | 0.688 | 3.000 | 1.500 |
| 41 | ORS598 |  | 300-600 | 4 | 4 | 100 | 0.371 | 1.484 | 2.583 | 0.646 |
| 42 | ORS613 |  | 280-290 | 2 | 2 | 100 | 0.493 | 0.985 | 2.208 | 1.104 |
| 43 | ORS620 |  | 200-420 | 2 | 2 | 100 | 0.298 | 0.595 | 3.083 | 1.542 |
| 44 | ORS621 |  | 340-980 | 6 | 6 | 100 | 0.232 | 1.392 | 5.083 | 0.847 |
| 45 | ORS628 |  | 410-430 | 2 | 2 | 100 | 0.373 | 0.746 | 1.375 | 0.688 |
| 46 | ORS630 |  | 320-640 | 4 | 4 | 100 | 0.135 | 0.675 | 2.375 | 0.475 |
| 47 | ORS637 |  | 180-320 | 3 | 3 | 100 | 0.289 | 0.868 | 1.167 | 0.389 |
| 48 | ORS650 |  | 160-550 | 3 | 3 | 100 | 0.272 | 0.817 | 1.042 | 0.347 |
| 49 | ORS669 |  | 180-430 | 4 | 4 | 100 | 0.237 | 0.948 | 4.917 | 1.229 |
| 50 | ORS674 |  | 230-430 | 3 | 3 | 100 | 0.297 | 0.891 | 2.042 | 0.681 |
| 51 | ORS684 |  | 200-300 | 2 | 2 | 100 | 0.153 | 0.306 | 2.000 | 1.000 |
| 52 | ORS686 |  | 320-520 | 4 | 4 | 100 | 0.349 | 1.395 | 3.375 | 0.844 |
| 53 | ORS694 |  | 160-500 | 3 | 3 | 100 | 0.324 | 0.971 | 2.542 | 0.847 |
| 54 | ORS706 |  | 160-320 | 4 | 4 | 100 | 0.377 | 1.510 | 3.708 | 0.927 |
| 55 | ORS707 |  | 160-180 | 2 | 2 | 100 | 0.287 | 0.575 | 2.583 | 1.292 |
| 56 | ORS725 |  | 280-720 | 4 | 4 | 100 | 0.484 | 1.936 | 4.250 | 1.063 |
| 57 | ORS727 |  | 160-180 | 2 | 2 | 100 | 0.274 | 0.549 | 2.667 | 1.333 |
| 58 | ORS739 |  | 140-180 | 3 | 3 | 100 | 0.223 | 0.669 | 4.042 | 1.347 |
| 59 | ORS762 |  | 510-650 | 4 | 4 | 100 | 0.465 | 1.859 | 3.500 | 0.875 |
| 60 | ORS805 |  | 160-450 | 2 | 2 | 100 | 0.232 | 0.464 | 1.875 | 0.938 |
| 61 | ORS807 |  | 220-240 | 2 | 2 | 100 | 0.444 | 0.889 | 2.667 | 1.333 |
| 62 | ORS812 |  | 150-550 | 3 | 3 | 100 | 0.115 | 0.346 | 1.958 | 0.653 |
| 63 | ORS817 |  | 180-260 | 3 | 3 | 100 | 0.117 | 0.350 | 2.042 | 0.681 |
| 64 | ORS822 |  | 180-320 | 3 | 3 | 100 | 0.314 | 0.941 | 2.250 | 0.750 |
| 65 | ORS853 |  | 250-260 | 2 | 2 | 100 | 0.079 | 0.158 | 1.917 | 0.958 |
| 66 | ORS887 |  | 290-630 | 4 | 4 | 100 | 0.286 | 1.146 | 5.667 | 1.417 |
| 67 | ORS899 |  | 140-420 | 2 | 2 | 100 | 0.197 | 0.395 | 2.208 | 1.104 |
| 68 | ORS902 |  | 140-240 | 3 | 3 | 100 | 0.371 | 1.113 | 2.250 | 0.750 |
| 69 | ORS924 |  | 300-320 | 2 | 2 | 100 | 0.288 | 0.576 | 2.833 | 1.417 |
| 70 | ORS926 |  | 160-180 | 2 | 2 | 100 | 0.383 | 0.766 | 2.125 | 1.063 |
| 71 | ORS928 |  | 520-580 | 3 | 3 | 100 | 0.428 | 1.285 | 2.500 | 0.833 |
| 72 | ORS930 |  | 220-380 | 2 | 2 | 100 | 0.286 | 0.572 | 1.208 | 0.604 |
| 73 | ORS935 |  | 460-620 | 3 | 3 | 100 | 0.316 | 0.947 | 2.292 | 0.764 |
| 74 | ORS962 |  | 220-600 | 3 | 3 | 100 | 0.440 | 1.319 | 2.625 | 0.875 |
| 75 | ORS965 |  | 180-200 | 2 | 2 | 100 | 0.270 | 0.540 | 2.250 | 1.125 |
| 76 | ORS983 |  | 280-320 | 2 | 2 | 100 | 0.179 | 0.358 | 2.250 | 1.125 |
| 77 | ORS990 |  | 140-150 | 2 | 2 | 100 | 0.201 | 0.402 | 3.542 | 1.771 |
| 78 | ORS1024 |  | 200-220 | 2 | 2 | 100 | 0.373 | 0.746 | 1.208 | 0.604 |
| 79 | ORS1030 |  | 360-380 | 2 | 2 | 100 | 0.164 | 0.329 | 1.792 | 0.896 |
| 80 | ORS1040 |  | 140-160 | 2 | 2 | 100 | 0.234 | 0.468 | 1.958 | 0.979 |
| 81 | ORS1043 |  | 160-200 | 3 | 3 | 100 | 0.386 | 1.158 | 3.333 | 1.111 |
| 82 | ORS1064 |  | 280-560 | 4 | 4 | 100 | 0.480 | 1.922 | 4.333 | 1.083 |
| 83 | ORS1068 |  | 360-380 | 2 | 2 | 100 | 0.421 | 0.843 | 2.042 | 1.021 |
| 84 | ORS1073 |  | 140-420 | 3 | 3 | 100 | 0.328 | 0.984 | 1.708 | 0.569 |
| 85 | ORS1080 |  | 220-230 | 2 | 2 | 100 | 0.305 | 0.609 | 2.000 | 1.000 |
| 86 | ORS1085 |  | 280-300 | 2 | 2 | 100 | 0.345 | 0.691 | 0.917 | 0.458 |
| 87 | ORS1086 |  | 160-180 | 2 | 2 | 100 | 0.300 | 0.600 | 2.375 | 1.188 |
| 88 | ORS1097 |  | 110-450 | 4 | 4 | 100 | 0.318 | 1.273 | 4.042 | 1.010 |
| 89 | ORS1114 |  | 320-330 | 2 | 2 | 100 | 0.387 | 0.773 | 1.625 | 0.813 |
| 90 | ORS1120 |  | 140-320 | 2 | 2 | 100 | 0.079 | 0.158 | 0.167 | 0.083 |
| 91 | ORS1130 |  | 290-300 | 2 | 2 | 100 | 0.187 | 0.373 | 2.000 | 1.000 |
| 92 | ORS1152 |  | 200-220 | 2 | 2 | 100 | 0.278 | 0.556 | 2.000 | 1.000 |
| 93 | ORS1159 |  | 310-320 | 2 | 2 | 100 | 0.149 | 0.299 | 1.833 | 0.917 |
| 94 | ORS1190 |  | 350-600 | 3 | 3 | 100 | 0.381 | 1.144 | 2.917 | 0.972 |
| 95 | ORS1193 |  | 220-230 | 2 | 2 | 100 | 0.219 | 0.438 | 2.000 | 1.000 |
| 96 | ORS1197 |  | 180-190 | 2 | 2 | 100 | 0.364 | 0.727 | 1.500 | 0.750 |
| 97 | ORS1219 |  | 200-400 | 3 | 3 | 100 | 0.259 | 0.777 | 2.625 | 0.875 |
| 98 | ORS1242 |  | 290-300 | 2 | 2 | 100 | 0.374 | 0.748 | 1.917 | 0.958 |
| 99 | ORS1245 |  | 240-250 | 2 | 2 | 100 | 0.135 | 0.270 | 1.958 | 0.979 |
| 100 | ORS1259 |  | 160-380 | 2 | 2 | 100 | 0.152 | 0.304 | 2.083 | 1.042 |
| 101 | ORS1264 |  | 200-300 | 3 | 3 | 100 | 0.254 | 0.763 | 1.208 | 0.403 |
| 102 | ORS1265 |  | 190-220 | 2 | 2 | 100 | 0.430 | 0.859 | 2.000 | 1.000 |
| 103 | ORS1271 |  | 190-200 | 2 | 2 | 100 | 0.347 | 0.694 | 2.375 | 1.188 |
|  |  |  |  | 267 | 267 |  | 0.301 | 0.789 | 2.331 | 0.919 |

TB: Total Bands, PB: Polymorphic Bands, %P: Percentage Of Polymorphism, Avg PIC: Average Polymorphic Information Content, PI: Primer Index, (Rp):Resolving Power
