# Supplementary material for: Assessing population structure and morpho-molecular characterization of sunflower (Helianthus annuus L.) for elite germplasm identification
Source: PeerJ. 2024 Oct 31;12:e18205. doi: 10.7717/peerj.18205 (PMC11531741; doi:10.7717/peerj.18205)
Supplement: Table S3 [file peerj-12-18205-s005.docx]

Supplementary Table 3: Molecular clustering based neighbor joining method with Jaccard’s pairwise distance matrix, based on SSR marker data from 48 sunflower germplasm accessions.

| **S.no** | **clusters** | **No of genotypes** | **List of genotypes** |
| --- | --- | --- | --- |
| **1** | I | 12 | GMU 477, HOCL 6R, RHA GPR 110, IB 80, CMS 107B, PM 36, IL 77, RHA95-C-10, IL84, GMU 755, GMU 325, GP6 1089 |
| **2** | II | 7 | GMU 344, CSFI 99 ©, RHA 272-1, GMU 741, CMS 108B, COSFV 5, GMU 734 |
| **3** | III | 14 | ARM 248B, COSF 6B ©, GMU 1181, RHA 102, CMS 1103B, GMU 780, CMS 335B, RCR 72, CMSNDCMS2B, RHA GPR 58, GMU 450, PM 95, GMU 428, GMU 411 |
| **4** | IV | 5 | GMU 336, CMS 911B, ARM 240B, CMS 597B, PM 53 |
| **5** | V | 10 | RHA 278, RHA 273, RHA 857, GP6 912, CMS 135B, REC 431, RHA 378, PM 65, RHA GMU 755, COSF 13B |
