# Supplementary material for: Assessing population structure and morpho-molecular characterization of sunflower (Helianthus annuus L.) for elite germplasm identification
Source: PeerJ. 2024 Oct 31;12:e18205. doi: 10.7717/peerj.18205 (PMC11531741; doi:10.7717/peerj.18205)
Supplement: Table S4 — The genotypes are follows as: 1. ARM 248B, 2. COSF 6B ©, 3. GMU 1181, 4. RHA 102, 5. CMS 1103B, 6. GMU 780, 7. CMS 335B, 8. RCR 72, 9. CMSNDCMS2B, 10. GMU 344, 11. CSFI 99 ©, 12. RHA 272-1, 13. GMU 336, 14. RHA GPR 58, 15. CMS 911B, 16. GMU 477, 17. GMU 450, 18. PM 95, 19. GMU 428, 20. GMU 411, 21. GMU 741, 22. CMS 108B, 23. COSFV ©5, 24. GMU 734, 25. ARM 240B, 26. CMS 597B, 27. HOCL 6R, 28. RHA GPR 110, 29. IB 80, 30. CMS 107B, 31. PM 36, 32. IL 77, 33. RHA95-C-10, 34. IL84, 35. RHA 278, 36. RHA 273, 37. RHA 857, 38. GMU 755, 39. PM 53, 40. GP6 912, 41. CMS 135B, 42. REC 431, 43. GMU 325, 44. GP6 1089, 45. RHA 378, 46. PM 65, 47. RHA GMU 755 and 48. COSF 13B. [file peerj-12-18205-s006.docx]

Supplementary Table 4: Jaccard Dissimilarity index of 48 genotypes of sunflower

| 48 | 1 | 2 | 3 | 4 | 5 | 6 | 7 | 8 | 9 | 10 | 11 | 12 | 13 | 14 | 15 | 16 | 17 | 18 | 19 | 20 | 21 | 22 | 23 | 24 | 25 | 26 | 27 | 28 | 29 | 30 | 31 | 32 | 33 | 34 | 35 | 36 | 37 | 38 | 39 | 40 | 41 | 42 | 43 | 44 | 45 | 46 | 47 | 48 |
| --- | --- | --- | --- | --- | --- | --- | --- | --- | --- | --- | --- | --- | --- | --- | --- | --- | --- | --- | --- | --- | --- | --- | --- | --- | --- | --- | --- | --- | --- | --- | --- | --- | --- | --- | --- | --- | --- | --- | --- | --- | --- | --- | --- | --- | --- | --- | --- | --- |
| 1 | 1 |  |  |  |  |  |  |  |  |  |  |  |  |  |  |  |  |  |  |  |  |  |  |  |  |  |  |  |  |  |  |  |  |  |  |  |  |  |  |  |  |  |  |  |  |  |  |  |
| 2 | 0.48 | 1.00 |  |  |  |  |  |  |  |  |  |  |  |  |  |  |  |  |  |  |  |  |  |  |  |  |  |  |  |  |  |  |  |  |  |  |  |  |  |  |  |  |  |  |  |  |  |  |
| 3 | 0.49 | 0.47 | 1.00 |  |  |  |  |  |  |  |  |  |  |  |  |  |  |  |  |  |  |  |  |  |  |  |  |  |  |  |  |  |  |  |  |  |  |  |  |  |  |  |  |  |  |  |  |  |
| 4 | 0.48 | 0.46 | 0.41 | 1.00 |  |  |  |  |  |  |  |  |  |  |  |  |  |  |  |  |  |  |  |  |  |  |  |  |  |  |  |  |  |  |  |  |  |  |  |  |  |  |  |  |  |  |  |  |
| 5 | 0.44 | 0.49 | 0.44 | 0.43 | 1.00 |  |  |  |  |  |  |  |  |  |  |  |  |  |  |  |  |  |  |  |  |  |  |  |  |  |  |  |  |  |  |  |  |  |  |  |  |  |  |  |  |  |  |  |
| 6 | 0.49 | 0.54 | 0.50 | 0.48 | 0.49 | 1.00 |  |  |  |  |  |  |  |  |  |  |  |  |  |  |  |  |  |  |  |  |  |  |  |  |  |  |  |  |  |  |  |  |  |  |  |  |  |  |  |  |  |  |
| 7 | 0.47 | 0.50 | 0.43 | 0.46 | 0.45 | 0.45 | 1.00 |  |  |  |  |  |  |  |  |  |  |  |  |  |  |  |  |  |  |  |  |  |  |  |  |  |  |  |  |  |  |  |  |  |  |  |  |  |  |  |  |  |
| 8 | 0.54 | 0.51 | 0.43 | 0.46 | 0.43 | 0.49 | 0.41 | 1.00 |  |  |  |  |  |  |  |  |  |  |  |  |  |  |  |  |  |  |  |  |  |  |  |  |  |  |  |  |  |  |  |  |  |  |  |  |  |  |  |  |
| 9 | 0.45 | 0.47 | 0.47 | 0.45 | 0.43 | 0.47 | 0.41 | 0.46 | 1.00 |  |  |  |  |  |  |  |  |  |  |  |  |  |  |  |  |  |  |  |  |  |  |  |  |  |  |  |  |  |  |  |  |  |  |  |  |  |  |  |
| 10 | 0.44 | 0.53 | 0.49 | 0.50 | 0.47 | 0.53 | 0.48 | 0.47 | 0.43 | 1.00 |  |  |  |  |  |  |  |  |  |  |  |  |  |  |  |  |  |  |  |  |  |  |  |  |  |  |  |  |  |  |  |  |  |  |  |  |  |  |
| 11 | 0.51 | 0.49 | 0.41 | 0.45 | 0.48 | 0.50 | 0.49 | 0.50 | 0.45 | 0.45 | 1.00 |  |  |  |  |  |  |  |  |  |  |  |  |  |  |  |  |  |  |  |  |  |  |  |  |  |  |  |  |  |  |  |  |  |  |  |  |  |
| 12 | 0.56 | 0.60 | 0.51 | 0.46 | 0.58 | 0.58 | 0.49 | 0.57 | 0.49 | 0.52 | 0.42 | 1.00 |  |  |  |  |  |  |  |  |  |  |  |  |  |  |  |  |  |  |  |  |  |  |  |  |  |  |  |  |  |  |  |  |  |  |  |  |
| 13 | 0.55 | 0.57 | 0.47 | 0.48 | 0.51 | 0.56 | 0.49 | 0.55 | 0.53 | 0.49 | 0.53 | 0.52 | 1.00 |  |  |  |  |  |  |  |  |  |  |  |  |  |  |  |  |  |  |  |  |  |  |  |  |  |  |  |  |  |  |  |  |  |  |  |
| 14 | 0.48 | 0.44 | 0.39 | 0.37 | 0.45 | 0.47 | 0.46 | 0.48 | 0.47 | 0.47 | 0.43 | 0.42 | 0.46 | 1.00 |  |  |  |  |  |  |  |  |  |  |  |  |  |  |  |  |  |  |  |  |  |  |  |  |  |  |  |  |  |  |  |  |  |  |
| 15 | 0.60 | 0.55 | 0.57 | 0.52 | 0.49 | 0.49 | 0.50 | 0.51 | 0.55 | 0.55 | 0.61 | 0.64 | 0.51 | 0.46 | 1.00 |  |  |  |  |  |  |  |  |  |  |  |  |  |  |  |  |  |  |  |  |  |  |  |  |  |  |  |  |  |  |  |  |  |
| 16 | 0.58 | 0.55 | 0.48 | 0.52 | 0.48 | 0.54 | 0.44 | 0.54 | 0.50 | 0.56 | 0.50 | 0.51 | 0.57 | 0.43 | 0.55 | 1.00 |  |  |  |  |  |  |  |  |  |  |  |  |  |  |  |  |  |  |  |  |  |  |  |  |  |  |  |  |  |  |  |  |
| 17 | 0.53 | 0.54 | 0.51 | 0.38 | 0.53 | 0.49 | 0.46 | 0.49 | 0.46 | 0.51 | 0.50 | 0.50 | 0.51 | 0.40 | 0.49 | 0.48 | 1.00 |  |  |  |  |  |  |  |  |  |  |  |  |  |  |  |  |  |  |  |  |  |  |  |  |  |  |  |  |  |  |  |
| 18 | 0.45 | 0.54 | 0.51 | 0.47 | 0.50 | 0.53 | 0.46 | 0.47 | 0.48 | 0.49 | 0.50 | 0.57 | 0.45 | 0.46 | 0.57 | 0.51 | 0.37 | 1.00 |  |  |  |  |  |  |  |  |  |  |  |  |  |  |  |  |  |  |  |  |  |  |  |  |  |  |  |  |  |  |
| 19 | 0.47 | 0.52 | 0.36 | 0.44 | 0.49 | 0.45 | 0.43 | 0.40 | 0.41 | 0.47 | 0.42 | 0.49 | 0.52 | 0.42 | 0.53 | 0.50 | 0.40 | 0.35 | 1.00 |  |  |  |  |  |  |  |  |  |  |  |  |  |  |  |  |  |  |  |  |  |  |  |  |  |  |  |  |  |
| 20 | 0.43 | 0.50 | 0.43 | 0.45 | 0.45 | 0.38 | 0.37 | 0.49 | 0.37 | 0.43 | 0.48 | 0.48 | 0.48 | 0.43 | 0.46 | 0.48 | 0.38 | 0.41 | 0.34 | 1.00 |  |  |  |  |  |  |  |  |  |  |  |  |  |  |  |  |  |  |  |  |  |  |  |  |  |  |  |  |
| 21 | 0.53 | 0.54 | 0.48 | 0.49 | 0.48 | 0.48 | 0.46 | 0.44 | 0.45 | 0.43 | 0.42 | 0.46 | 0.45 | 0.46 | 0.56 | 0.55 | 0.44 | 0.48 | 0.45 | 0.40 | 1.00 |  |  |  |  |  |  |  |  |  |  |  |  |  |  |  |  |  |  |  |  |  |  |  |  |  |  |  |
| 22 | 0.58 | 0.57 | 0.56 | 0.53 | 0.54 | 0.54 | 0.53 | 0.57 | 0.47 | 0.48 | 0.48 | 0.52 | 0.57 | 0.52 | 0.62 | 0.55 | 0.47 | 0.55 | 0.50 | 0.48 | 0.42 | 1.00 |  |  |  |  |  |  |  |  |  |  |  |  |  |  |  |  |  |  |  |  |  |  |  |  |  |  |
| 23 | 0.59 | 0.55 | 0.51 | 0.53 | 0.55 | 0.53 | 0.51 | 0.57 | 0.48 | 0.49 | 0.47 | 0.47 | 0.51 | 0.41 | 0.51 | 0.53 | 0.43 | 0.48 | 0.46 | 0.43 | 0.45 | 0.47 | 1.00 |  |  |  |  |  |  |  |  |  |  |  |  |  |  |  |  |  |  |  |  |  |  |  |  |  |
| 24 | 0.56 | 0.61 | 0.56 | 0.51 | 0.59 | 0.54 | 0.57 | 0.60 | 0.54 | 0.54 | 0.54 | 0.40 | 0.56 | 0.47 | 0.63 | 0.57 | 0.51 | 0.51 | 0.53 | 0.49 | 0.53 | 0.52 | 0.47 | 1.00 |  |  |  |  |  |  |  |  |  |  |  |  |  |  |  |  |  |  |  |  |  |  |  |  |
| 25 | 0.46 | 0.60 | 0.52 | 0.49 | 0.55 | 0.57 | 0.58 | 0.61 | 0.51 | 0.56 | 0.50 | 0.54 | 0.53 | 0.54 | 0.62 | 0.56 | 0.50 | 0.51 | 0.52 | 0.53 | 0.54 | 0.60 | 0.60 | 0.57 | 1.00 |  |  |  |  |  |  |  |  |  |  |  |  |  |  |  |  |  |  |  |  |  |  |  |
| 26 | 0.52 | 0.49 | 0.47 | 0.45 | 0.52 | 0.49 | 0.51 | 0.52 | 0.50 | 0.53 | 0.51 | 0.50 | 0.51 | 0.48 | 0.52 | 0.60 | 0.47 | 0.55 | 0.45 | 0.46 | 0.52 | 0.53 | 0.51 | 0.53 | 0.43 | 1.00 |  |  |  |  |  |  |  |  |  |  |  |  |  |  |  |  |  |  |  |  |  |  |
| 27 | 0.48 | 0.54 | 0.42 | 0.35 | 0.50 | 0.53 | 0.47 | 0.51 | 0.46 | 0.50 | 0.41 | 0.42 | 0.50 | 0.41 | 0.58 | 0.50 | 0.41 | 0.46 | 0.43 | 0.45 | 0.48 | 0.51 | 0.47 | 0.48 | 0.47 | 0.43 | 1.00 |  |  |  |  |  |  |  |  |  |  |  |  |  |  |  |  |  |  |  |  |  |
| 28 | 0.52 | 0.52 | 0.42 | 0.37 | 0.51 | 0.46 | 0.47 | 0.52 | 0.50 | 0.56 | 0.54 | 0.49 | 0.54 | 0.40 | 0.55 | 0.46 | 0.41 | 0.53 | 0.45 | 0.44 | 0.50 | 0.50 | 0.51 | 0.51 | 0.56 | 0.47 | 0.39 | 1.00 |  |  |  |  |  |  |  |  |  |  |  |  |  |  |  |  |  |  |  |  |
| 29 | 0.55 | 0.61 | 0.51 | 0.45 | 0.48 | 0.53 | 0.54 | 0.51 | 0.52 | 0.55 | 0.51 | 0.55 | 0.62 | 0.49 | 0.59 | 0.54 | 0.48 | 0.50 | 0.50 | 0.49 | 0.49 | 0.54 | 0.52 | 0.54 | 0.51 | 0.51 | 0.43 | 0.38 | 1.00 |  |  |  |  |  |  |  |  |  |  |  |  |  |  |  |  |  |  |  |
| 30 | 0.46 | 0.56 | 0.52 | 0.42 | 0.50 | 0.44 | 0.49 | 0.51 | 0.49 | 0.51 | 0.45 | 0.50 | 0.53 | 0.45 | 0.58 | 0.55 | 0.41 | 0.46 | 0.44 | 0.45 | 0.49 | 0.52 | 0.46 | 0.50 | 0.45 | 0.41 | 0.33 | 0.44 | 0.41 | 1.00 |  |  |  |  |  |  |  |  |  |  |  |  |  |  |  |  |  |  |
| 31 | 0.56 | 0.61 | 0.52 | 0.52 | 0.54 | 0.52 | 0.47 | 0.53 | 0.54 | 0.59 | 0.47 | 0.47 | 0.59 | 0.53 | 0.60 | 0.52 | 0.50 | 0.51 | 0.45 | 0.46 | 0.46 | 0.49 | 0.50 | 0.49 | 0.50 | 0.49 | 0.45 | 0.45 | 0.46 | 0.41 | 1.00 |  |  |  |  |  |  |  |  |  |  |  |  |  |  |  |  |  |
| 32 | 0.53 | 0.57 | 0.51 | 0.46 | 0.51 | 0.49 | 0.50 | 0.49 | 0.48 | 0.53 | 0.49 | 0.49 | 0.53 | 0.50 | 0.54 | 0.54 | 0.42 | 0.47 | 0.44 | 0.40 | 0.42 | 0.49 | 0.46 | 0.48 | 0.50 | 0.39 | 0.37 | 0.40 | 0.43 | 0.36 | 0.38 | 1.00 |  |  |  |  |  |  |  |  |  |  |  |  |  |  |  |  |
| 33 | 0.58 | 0.62 | 0.53 | 0.54 | 0.52 | 0.56 | 0.55 | 0.58 | 0.46 | 0.54 | 0.52 | 0.50 | 0.59 | 0.50 | 0.58 | 0.43 | 0.51 | 0.56 | 0.53 | 0.50 | 0.56 | 0.50 | 0.53 | 0.54 | 0.54 | 0.53 | 0.46 | 0.44 | 0.44 | 0.47 | 0.46 | 0.45 | 1.00 |  |  |  |  |  |  |  |  |  |  |  |  |  |  |  |
| 34 | 0.49 | 0.55 | 0.53 | 0.45 | 0.52 | 0.53 | 0.52 | 0.54 | 0.46 | 0.49 | 0.48 | 0.48 | 0.52 | 0.47 | 0.56 | 0.51 | 0.45 | 0.49 | 0.51 | 0.46 | 0.49 | 0.44 | 0.47 | 0.49 | 0.46 | 0.46 | 0.43 | 0.48 | 0.47 | 0.44 | 0.46 | 0.38 | 0.45 | 1.00 |  |  |  |  |  |  |  |  |  |  |  |  |  |  |
| 35 | 0.51 | 0.58 | 0.50 | 0.56 | 0.50 | 0.49 | 0.47 | 0.48 | 0.52 | 0.55 | 0.50 | 0.58 | 0.55 | 0.50 | 0.58 | 0.53 | 0.55 | 0.53 | 0.49 | 0.48 | 0.52 | 0.53 | 0.57 | 0.57 | 0.58 | 0.52 | 0.53 | 0.51 | 0.52 | 0.51 | 0.51 | 0.51 | 0.52 | 0.49 | 1.00 |  |  |  |  |  |  |  |  |  |  |  |  |  |
| 36 | 0.55 | 0.58 | 0.54 | 0.56 | 0.51 | 0.54 | 0.43 | 0.52 | 0.53 | 0.53 | 0.58 | 0.54 | 0.49 | 0.50 | 0.54 | 0.52 | 0.51 | 0.52 | 0.52 | 0.50 | 0.53 | 0.54 | 0.51 | 0.53 | 0.60 | 0.54 | 0.54 | 0.50 | 0.58 | 0.58 | 0.52 | 0.54 | 0.56 | 0.50 | 0.44 | 1.00 |  |  |  |  |  |  |  |  |  |  |  |  |
| 37 | 0.49 | 0.62 | 0.52 | 0.50 | 0.53 | 0.51 | 0.49 | 0.51 | 0.56 | 0.51 | 0.56 | 0.56 | 0.48 | 0.48 | 0.58 | 0.56 | 0.52 | 0.52 | 0.50 | 0.51 | 0.51 | 0.52 | 0.55 | 0.59 | 0.52 | 0.51 | 0.52 | 0.53 | 0.54 | 0.55 | 0.51 | 0.53 | 0.54 | 0.49 | 0.47 | 0.41 | 1.00 |  |  |  |  |  |  |  |  |  |  |  |
| 38 | 0.52 | 0.54 | 0.51 | 0.54 | 0.52 | 0.57 | 0.50 | 0.53 | 0.50 | 0.54 | 0.53 | 0.53 | 0.50 | 0.44 | 0.55 | 0.47 | 0.50 | 0.46 | 0.50 | 0.47 | 0.50 | 0.57 | 0.49 | 0.52 | 0.54 | 0.49 | 0.45 | 0.49 | 0.54 | 0.48 | 0.46 | 0.49 | 0.45 | 0.43 | 0.52 | 0.46 | 0.51 | 1.00 |  |  |  |  |  |  |  |  |  |  |
| 39 | 0.55 | 0.58 | 0.54 | 0.50 | 0.44 | 0.51 | 0.49 | 0.45 | 0.57 | 0.51 | 0.55 | 0.61 | 0.58 | 0.50 | 0.50 | 0.53 | 0.51 | 0.51 | 0.49 | 0.52 | 0.52 | 0.54 | 0.53 | 0.63 | 0.57 | 0.52 | 0.51 | 0.51 | 0.51 | 0.47 | 0.51 | 0.53 | 0.56 | 0.55 | 0.52 | 0.53 | 0.50 | 0.49 | 1.00 |  |  |  |  |  |  |  |  |  |
| 40 | 0.57 | 0.55 | 0.50 | 0.46 | 0.49 | 0.52 | 0.51 | 0.49 | 0.52 | 0.55 | 0.51 | 0.58 | 0.52 | 0.46 | 0.56 | 0.45 | 0.49 | 0.51 | 0.49 | 0.53 | 0.48 | 0.55 | 0.50 | 0.56 | 0.53 | 0.50 | 0.49 | 0.48 | 0.49 | 0.47 | 0.46 | 0.48 | 0.49 | 0.50 | 0.48 | 0.49 | 0.46 | 0.46 | 0.44 | 1.00 |  |  |  |  |  |  |  |  |
| 41 | 0.55 | 0.56 | 0.54 | 0.48 | 0.54 | 0.51 | 0.52 | 0.55 | 0.51 | 0.53 | 0.53 | 0.58 | 0.53 | 0.50 | 0.52 | 0.60 | 0.47 | 0.53 | 0.47 | 0.49 | 0.54 | 0.54 | 0.50 | 0.58 | 0.53 | 0.47 | 0.49 | 0.51 | 0.51 | 0.42 | 0.51 | 0.48 | 0.53 | 0.53 | 0.50 | 0.53 | 0.48 | 0.50 | 0.47 | 0.41 | 1.00 |  |  |  |  |  |  |  |
| 42 | 0.47 | 0.55 | 0.53 | 0.49 | 0.50 | 0.54 | 0.50 | 0.54 | 0.51 | 0.54 | 0.54 | 0.59 | 0.54 | 0.50 | 0.54 | 0.58 | 0.47 | 0.47 | 0.49 | 0.48 | 0.53 | 0.54 | 0.52 | 0.58 | 0.50 | 0.54 | 0.47 | 0.53 | 0.52 | 0.47 | 0.53 | 0.49 | 0.58 | 0.50 | 0.46 | 0.51 | 0.48 | 0.56 | 0.52 | 0.47 | 0.41 | 1.00 |  |  |  |  |  |  |
| 43 | 0.58 | 0.56 | 0.51 | 0.50 | 0.47 | 0.59 | 0.54 | 0.54 | 0.50 | 0.58 | 0.47 | 0.53 | 0.57 | 0.49 | 0.54 | 0.52 | 0.47 | 0.54 | 0.48 | 0.52 | 0.51 | 0.52 | 0.49 | 0.52 | 0.54 | 0.50 | 0.49 | 0.47 | 0.46 | 0.47 | 0.45 | 0.43 | 0.42 | 0.44 | 0.47 | 0.51 | 0.55 | 0.41 | 0.50 | 0.41 | 0.43 | 0.47 | 1.00 |  |  |  |  |  |
| 44 | 0.62 | 0.61 | 0.53 | 0.54 | 0.52 | 0.57 | 0.56 | 0.58 | 0.55 | 0.59 | 0.57 | 0.56 | 0.56 | 0.53 | 0.58 | 0.54 | 0.52 | 0.54 | 0.52 | 0.48 | 0.48 | 0.57 | 0.52 | 0.58 | 0.58 | 0.56 | 0.52 | 0.51 | 0.54 | 0.54 | 0.51 | 0.53 | 0.50 | 0.48 | 0.56 | 0.54 | 0.57 | 0.48 | 0.59 | 0.49 | 0.53 | 0.52 | 0.44 | 1.00 |  |  |  |  |
| 45 | 0.56 | 0.57 | 0.48 | 0.45 | 0.47 | 0.52 | 0.48 | 0.46 | 0.47 | 0.54 | 0.50 | 0.50 | 0.51 | 0.48 | 0.56 | 0.54 | 0.49 | 0.54 | 0.47 | 0.45 | 0.44 | 0.56 | 0.50 | 0.58 | 0.53 | 0.48 | 0.50 | 0.49 | 0.49 | 0.49 | 0.49 | 0.49 | 0.53 | 0.48 | 0.48 | 0.49 | 0.45 | 0.47 | 0.53 | 0.44 | 0.46 | 0.48 | 0.45 | 0.51 | 1.00 |  |  |  |
| 46 | 0.54 | 0.57 | 0.55 | 0.51 | 0.53 | 0.55 | 0.52 | 0.55 | 0.51 | 0.51 | 0.54 | 0.57 | 0.58 | 0.54 | 0.56 | 0.56 | 0.53 | 0.53 | 0.46 | 0.49 | 0.54 | 0.48 | 0.54 | 0.57 | 0.55 | 0.47 | 0.56 | 0.53 | 0.52 | 0.52 | 0.50 | 0.53 | 0.54 | 0.47 | 0.51 | 0.49 | 0.48 | 0.51 | 0.50 | 0.47 | 0.45 | 0.49 | 0.51 | 0.52 | 0.49 | 1.00 |  |  |
| 47 | 0.61 | 0.59 | 0.57 | 0.60 | 0.57 | 0.59 | 0.56 | 0.56 | 0.54 | 0.57 | 0.55 | 0.56 | 0.54 | 0.54 | 0.57 | 0.62 | 0.56 | 0.58 | 0.54 | 0.54 | 0.56 | 0.57 | 0.49 | 0.62 | 0.61 | 0.50 | 0.56 | 0.60 | 0.59 | 0.53 | 0.53 | 0.55 | 0.59 | 0.53 | 0.53 | 0.50 | 0.53 | 0.52 | 0.57 | 0.49 | 0.44 | 0.54 | 0.49 | 0.56 | 0.48 | 0.44 | 1.00 |  |
| 48 | 0.54 | 0.56 | 0.54 | 0.56 | 0.56 | 0.55 | 0.57 | 0.52 | 0.56 | 0.58 | 0.58 | 0.65 | 0.60 | 0.53 | 0.57 | 0.59 | 0.56 | 0.53 | 0.54 | 0.54 | 0.60 | 0.63 | 0.60 | 0.57 | 0.58 | 0.53 | 0.58 | 0.62 | 0.63 | 0.56 | 0.59 | 0.57 | 0.65 | 0.58 | 0.52 | 0.56 | 0.54 | 0.56 | 0.57 | 0.51 | 0.52 | 0.50 | 0.52 | 0.56 | 0.50 | 0.57 | 0.51 | 1.00 |

(The genotypes are follows as: 1. ARM 248B, 2. COSF 6B ©, 3. GMU 1181, 4. RHA 102, 5. CMS 1103B, 6. GMU 780, 7. CMS 335B, 8. RCR 72, 9. CMSNDCMS2B, 10. GMU 344, 11. CSFI 99 ©, 12. RHA 272-1, 13. GMU 336, 14. RHA GPR 58, 15. CMS 911B, 16. GMU 477, 17. GMU 450, 18. PM 95, 19. GMU 428, 20. GMU 411, 21. GMU 741, 22. CMS 108B, 23. COSFV © 5, 24. GMU 734, 25. ARM 240B, 26. CMS 597B, 27. HOCL 6R, 28. RHA GPR 110, 29. IB 80, 30. CMS 107B, 31. PM 36, 32. IL 77, 33. RHA95-C-10, 34. IL84, 35. RHA 278, 36. RHA 273, 37. RHA 857, 38. GMU 755, 39. PM 53, 40. GP6 912, 41. CMS 135B, 42. REC 431, 43. GMU 325, 44. GP6 1089, 45. RHA 378, 46. PM 65, 47. RHA GMU 755 and 48. COSF 13B.)
