# Supplementary material for: Assessing population structure and morpho-molecular characterization of sunflower (Helianthus annuus L.) for elite germplasm identification
Source: PeerJ. 2024 Oct 31;12:e18205. doi: 10.7717/peerj.18205 (PMC11531741; doi:10.7717/peerj.18205)
Supplement: Table S6 [file peerj-12-18205-s008.docx]

Supplementary Table 6: Pairwise Population Matrix of Nei Unbiased Genetic Identity

| **S.no** | **Population** | **1** | **2** | **3** | **4** | **5** |
| --- | --- | --- | --- | --- | --- | --- |
| **1** | **I** | 1.000 |  |  |  |  |
| **2** | **II** | 0.965 | 1.000 |  |  |  |
| **3** | **III** | 0.965 | 0.977 | 1.000 |  |  |
| **4** | **IV** | 0.937 | 0.930 | 0.937 | 1.000 |  |
| **5** | **V** | 0.955 | 0.968 | 0.939 | 0.930 | 1.000 |
