# Supplementary material for: Assessing population structure and morpho-molecular characterization of sunflower (Helianthus annuus L.) for elite germplasm identification
Source: PeerJ. 2024 Oct 31;12:e18205. doi: 10.7717/peerj.18205 (PMC11531741; doi:10.7717/peerj.18205)
Supplement: Table S7 [file peerj-12-18205-s009.docx]

Supplementary Table 7: Forty-eight sunflower genotypes, including checks, were grouped into three hierarchical cluster based on PCs, eigenvalues exceeding 1, and Principal Factor Scores (PF scores) for different traits.

| **S.no** | **Clusters** | **No of genotypes** | **List of genotypes** |
| --- | --- | --- | --- |
| 1 | I | 19 | ARM 240B, CMS 108B, CMS 335B, CMS 597B, GMU 325, GMU 411, GMU 734, GMU 741, GP6 1089, IL 77, IL84, PM 53, PM 65, PM 95, RCR 72, RHA 272-1, RHA 273, RHA GMU 755, RHA95-C-10 |
| 2 | II | 15 | ARM 248B, CMS 1103B, CMS 911B, COSF 13B ©, COSF 6B ©, COSFV 5, CSFI 99 ©, GMU 1181, GMU 336, GMU 755  GMU 780, RHA 102, RHA 278, RHA GPR 110, RHA GPR 58 |
| 3 | III | 14 | CMS 107B, CMS 135B, CMSNDCMS2B, GMU 344, GMU 428, GMU 450, GMU 477, GP6 912, HOCL 6R, IB 80  PM 36, REC 431, RHA 378, RHA 857 |
