# Supplementary figures and images for: Assessing population structure and morpho-molecular characterization of sunflower (Helianthus annuus L.) for elite germplasm identification
Source: PeerJ. 2024 Oct 31;12:e18205. doi: 10.7717/peerj.18205 (PMC11531741; doi:10.7717/peerj.18205)

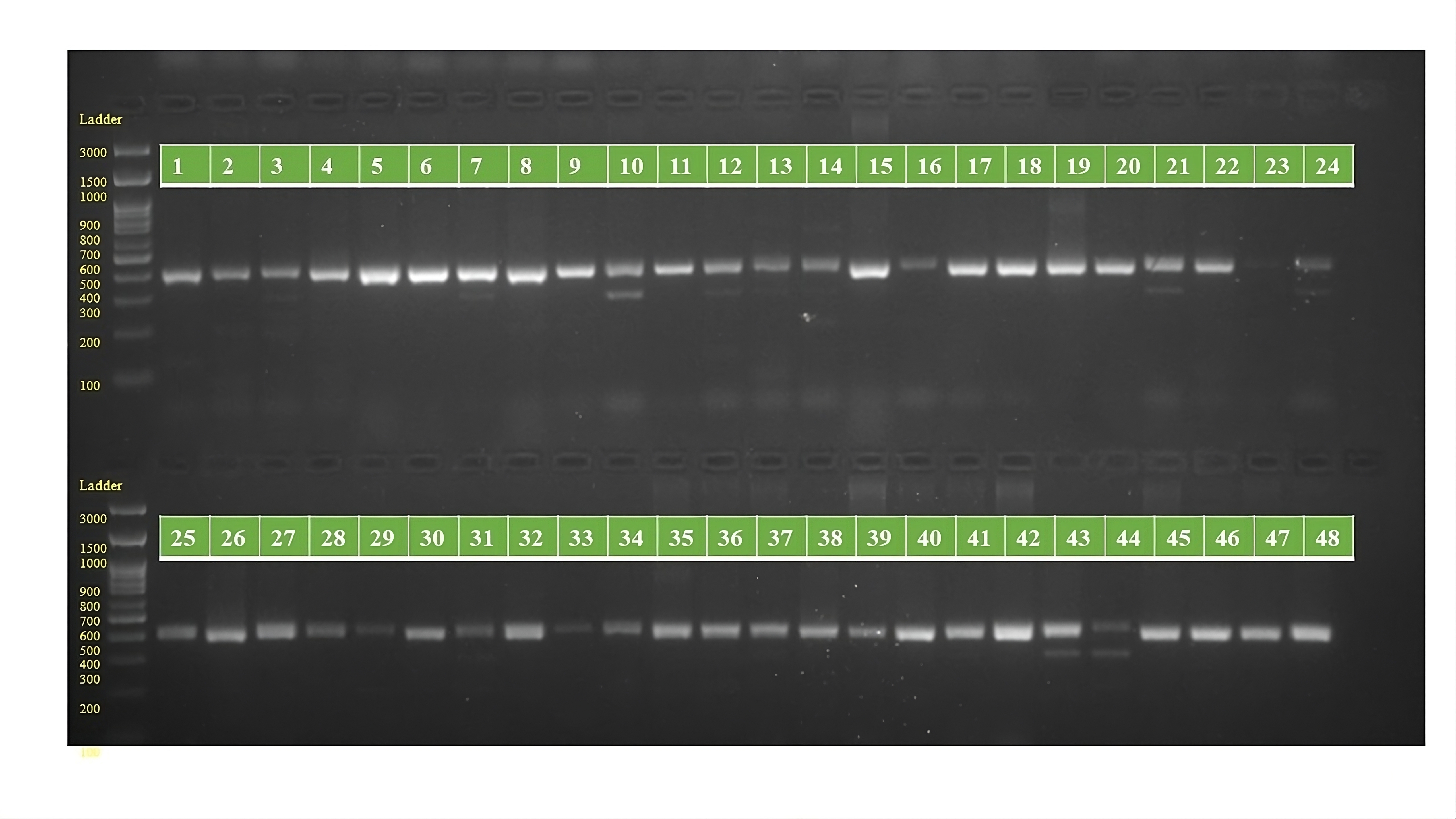

Supplement: Figure S1 — The genotypes are follows as: 1. ARM 248B, 2. COSF 6B ©, 3. GMU 1181, 4. RHA 102, 5. CMS 1103B, 6. GMU 780, 7. CMS 335B, 8. RCR 72, 9. CMSNDCMS2B, 10. GMU 344, 11. CSFI 99 ©, 12. RHA 272-1, 13. GMU 336, 14. RHA GPR 58, 15. CMS 911B, 16. GMU 477, 17. GMU 450, 18. PM 95, 19. GMU 428, 20. GMU 411, 21. GMU 741, 22. CMS 108B, 23. COSFV ©5, 24. GMU 734, 25. ARM 240B, 26. CMS 597B, 27. HOCL 6R, 28. RHA GPR 110, 29. IB 80, 30. CMS 107B, 31. PM 36, 32. IL 77, 33. RHA95-C-10, 34. IL84, 35. RHA 278, 36. RHA 273, 37. RHA 857, 38. GMU 755, 39. PM 53, 40. GP6 912, 41. CMS 135B, 42. REC 431, 43. GMU 325, 44. GP6 1089, 45. RHA 378, 46. PM 65, 47. RHA GMU 755 and 48. COSF 13B. [file peerj-12-18205-s010.jpg]

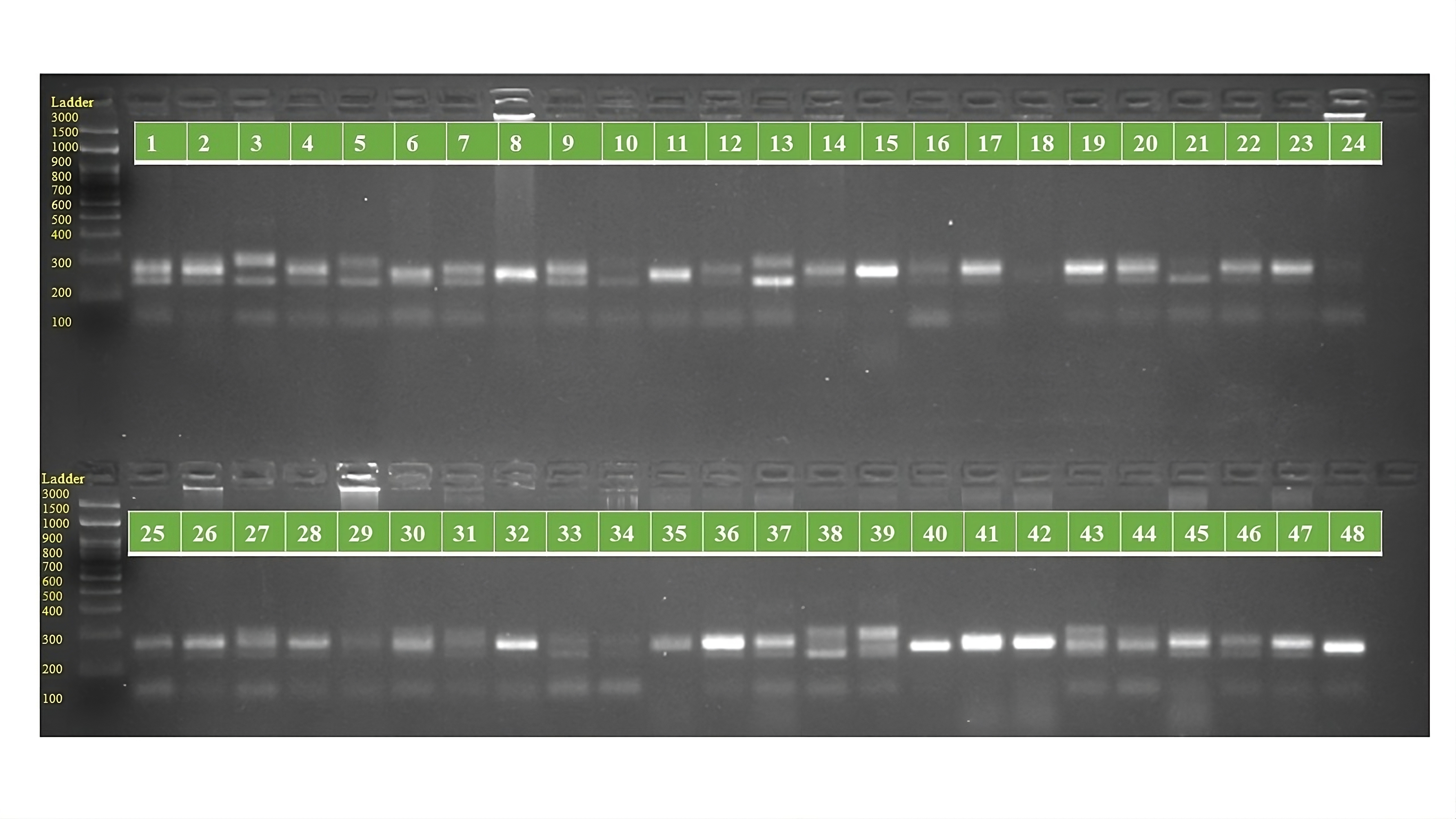

Supplement: Figure S2 — The genotypes are follows as: 1. ARM 248B, 2. COSF 6B ©, 3. GMU 1181, 4. RHA 102, 5. CMS 1103B, 6. GMU 780, 7. CMS 335B, 8. RCR 72, 9. CMSNDCMS2B, 10. GMU 344, 11. CSFI 99 ©, 12. RHA 272-1, 13. GMU 336, 14. RHA GPR 58, 15. CMS 911B, 16. GMU 477, 17. GMU 450, 18. PM 95, 19. GMU 428, 20. GMU 411, 21. GMU 741, 22. CMS 108B, 23. COSFV ©5, 24. GMU 734, 25. ARM 240B, 26. CMS 597B, 27. HOCL 6R, 28. RHA GPR 110, 29. IB 80, 30. CMS 107B, 31. PM 36, 32. IL 77, 33. RHA95-C-10, 34. IL84, 35. RHA 278, 36. RHA 273, 37. RHA 857, 38. GMU 755, 39. PM 53, 40. GP6 912, 41. CMS 135B, 42. REC 431, 43. GMU 325, 44. GP6 1089, 45. RHA 378, 46. PM 65, 47. RHA GMU 755 and 48. COSF 13B. [file peerj-12-18205-s011.jpg]

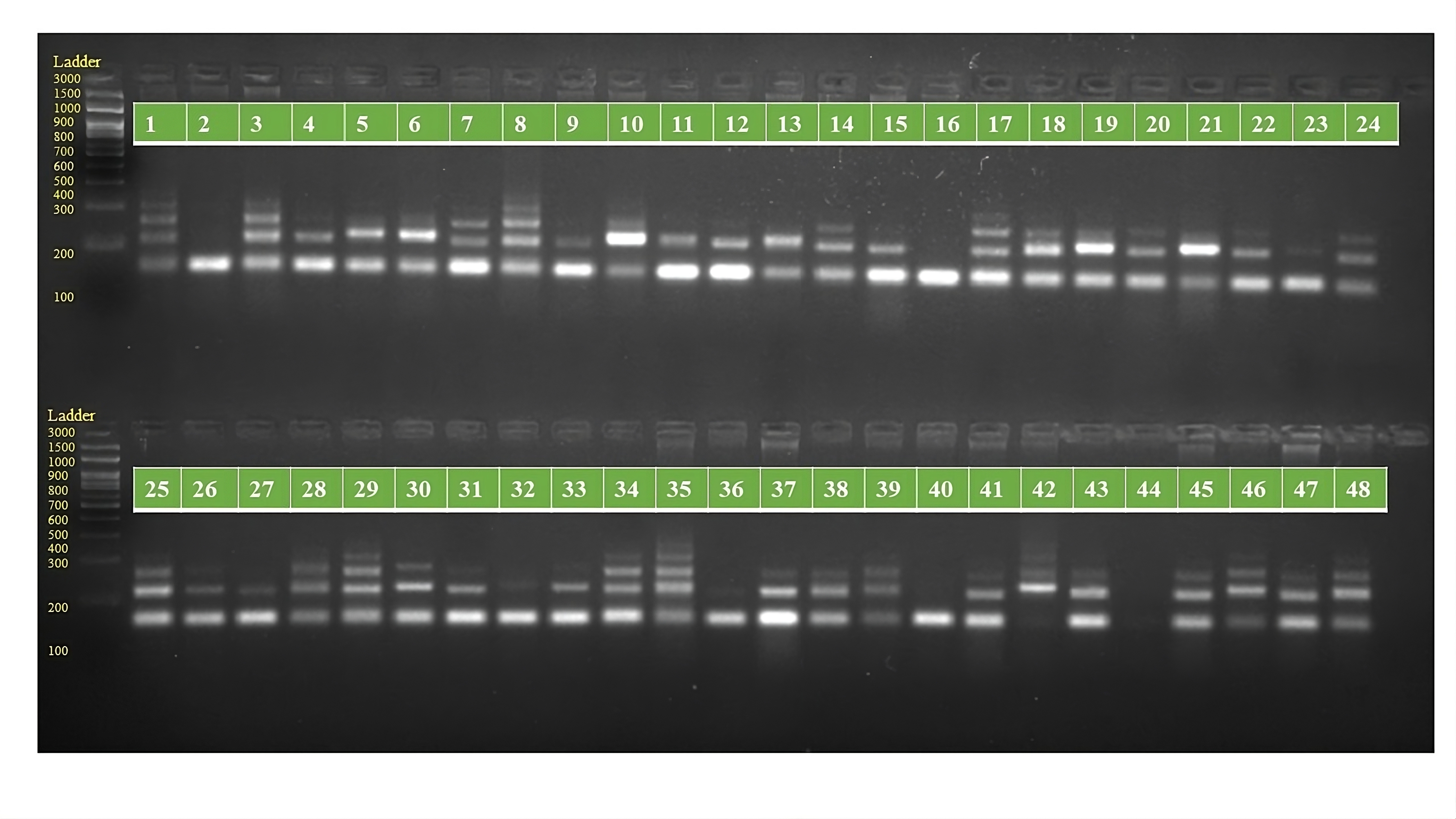

Supplement: Figure S3 — The genotypes are follows as: 1. ARM 248B, 2. COSF 6B ©, 3. GMU 1181, 4. RHA 102, 5. CMS 1103B, 6. GMU 780, 7. CMS 335B, 8. RCR 72, 9. CMSNDCMS2B, 10. GMU 344, 11. CSFI 99 ©, 12. RHA 272-1, 13. GMU 336, 14. RHA GPR 58, 15. CMS 911B, 16. GMU 477, 17. GMU 450, 18. PM 95, 19. GMU 428, 20. GMU 411, 21. GMU 741, 22. CMS 108B, 23. COSFV ©5, 24. GMU 734, 25. ARM 240B, 26. CMS 597B, 27. HOCL 6R, 28. RHA GPR 110, 29. IB 80, 30. CMS 107B, 31. PM 36, 32. IL 77, 33. RHA95-C-10, 34. IL84, 35. RHA 278, 36. RHA 273, 37. RHA 857, 38. GMU 755, 39. PM 53, 40. GP6 912, 41. CMS 135B, 42. REC 431, 43. GMU 325, 44. GP6 1089, 45. RHA 378, 46. PM 65, 47. RHA GMU 755 and 48. COSF 13B. [file peerj-12-18205-s012.jpg]

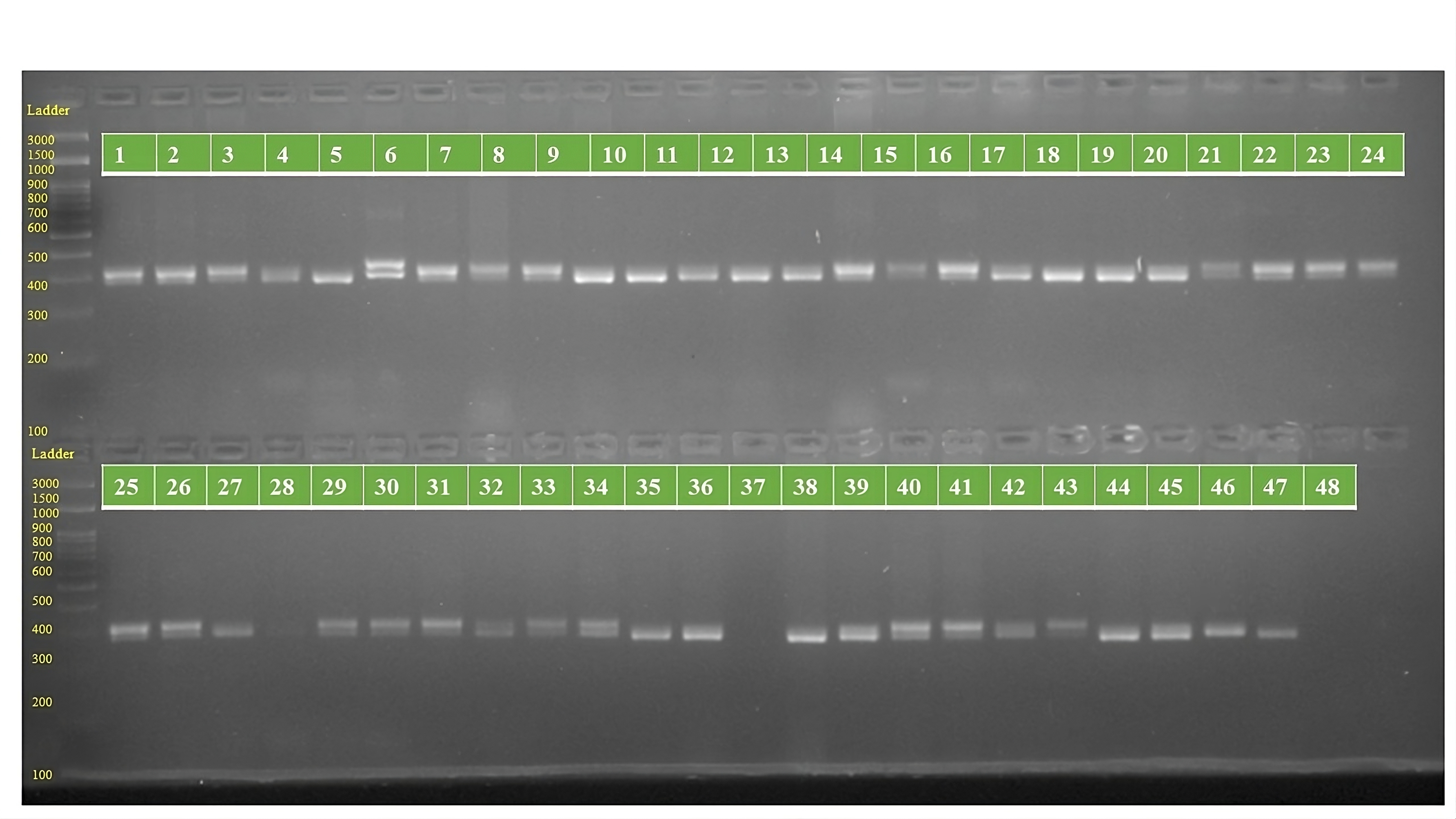

Supplement: Figure S4 — The genotypes are follows as: 1. ARM 248B, 2. COSF 6B ©, 3. GMU 1181, 4. RHA 102, 5. CMS 1103B, 6. GMU 780, 7. CMS 335B, 8. RCR 72, 9. CMSNDCMS2B, 10. GMU 344, 11. CSFI 99 ©, 12. RHA 272-1, 13. GMU 336, 14. RHA GPR 58, 15. CMS 911B, 16. GMU 477, 17. GMU 450, 18. PM 95, 19. GMU 428, 20. GMU 411, 21. GMU 741, 22. CMS 108B, 23. COSFV ©5, 24. GMU 734, 25. ARM 240B, 26. CMS 597B, 27. HOCL 6R, 28. RHA GPR 110, 29. IB 80, 30. CMS 107B, 31. PM 36, 32. IL 77, 33. RHA95-C-10, 34. IL84, 35. RHA 278, 36. RHA 273, 37. RHA 857, 38. GMU 755, 39. PM 53, 40. GP6 912, 41. CMS 135B, 42. REC 431, 43. GMU 325, 44. GP6 1089, 45. RHA 378, 46. PM 65, 47. RHA GMU 755 and 48. COSF 13B. [file peerj-12-18205-s013.jpg]

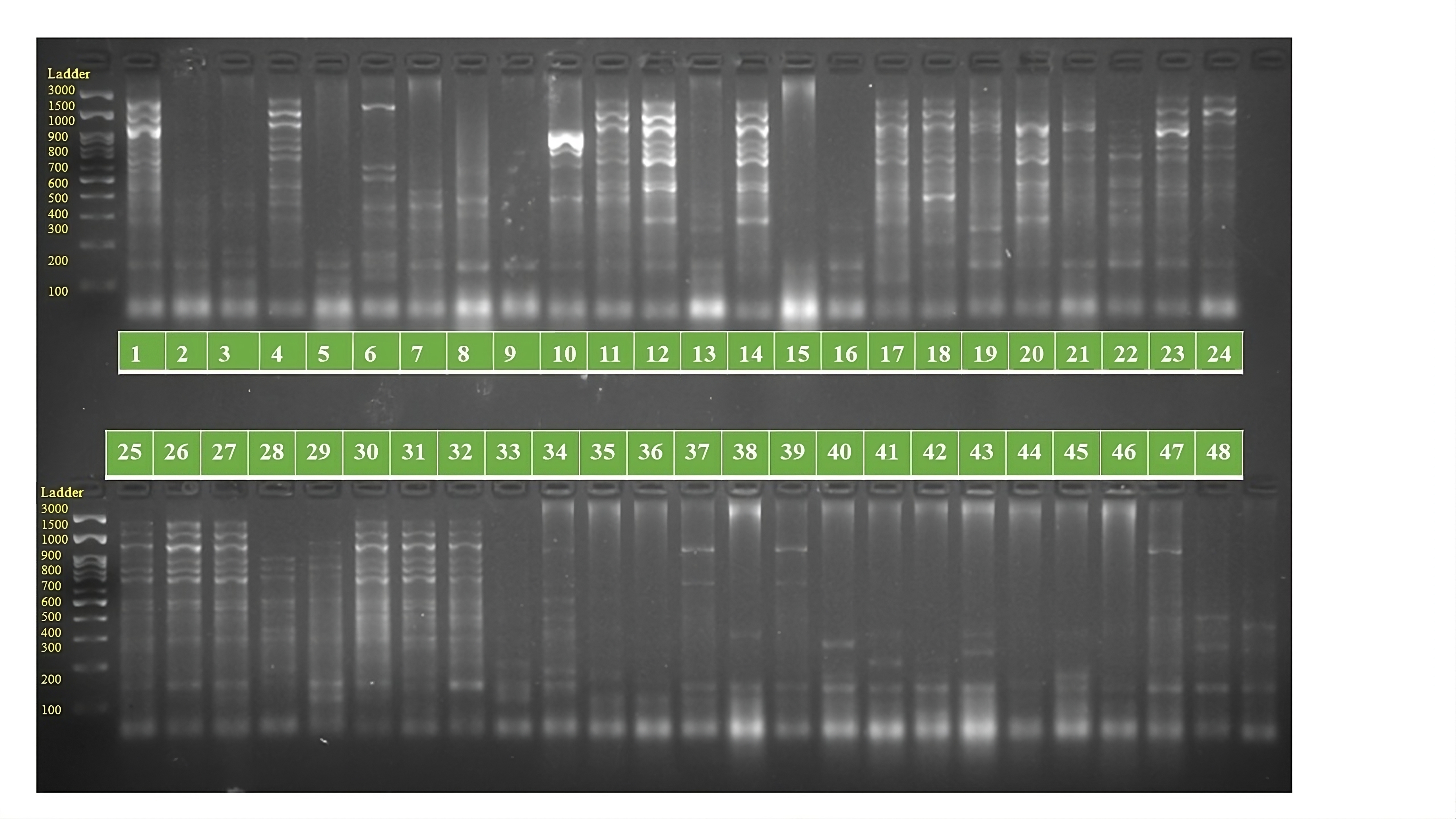

Supplement: Figure S5 — The genotypes are follows as: 1. ARM 248B, 2. COSF 6B ©, 3. GMU 1181, 4. RHA 102, 5. CMS 1103B, 6. GMU 780, 7. CMS 335B, 8. RCR 72, 9. CMSNDCMS2B, 10. GMU 344, 11. CSFI 99 ©, 12. RHA 272-1, 13. GMU 336, 14. RHA GPR 58, 15. CMS 911B, 16. GMU 477, 17. GMU 450, 18. PM 95, 19. GMU 428, 20. GMU 411, 21. GMU 741, 22. CMS 108B, 23. COSFV ©5, 24. GMU 734, 25. ARM 240B, 26. CMS 597B, 27. HOCL 6R, 28. RHA GPR 110, 29. IB 80, 30. CMS 107B, 31. PM 36, 32. IL 77, 33. RHA95-C-10, 34. IL84, 35. RHA 278, 36. RHA 273, 37. RHA 857, 38. GMU 755, 39. PM 53, 40. GP6 912, 41. CMS 135B, 42. REC 431, 43. GMU 325, 44. GP6 1089, 45. RHA 378, 46. PM 65, 47. RHA GMU 755 and 48. COSF 13B. [file peerj-12-18205-s014.jpg]

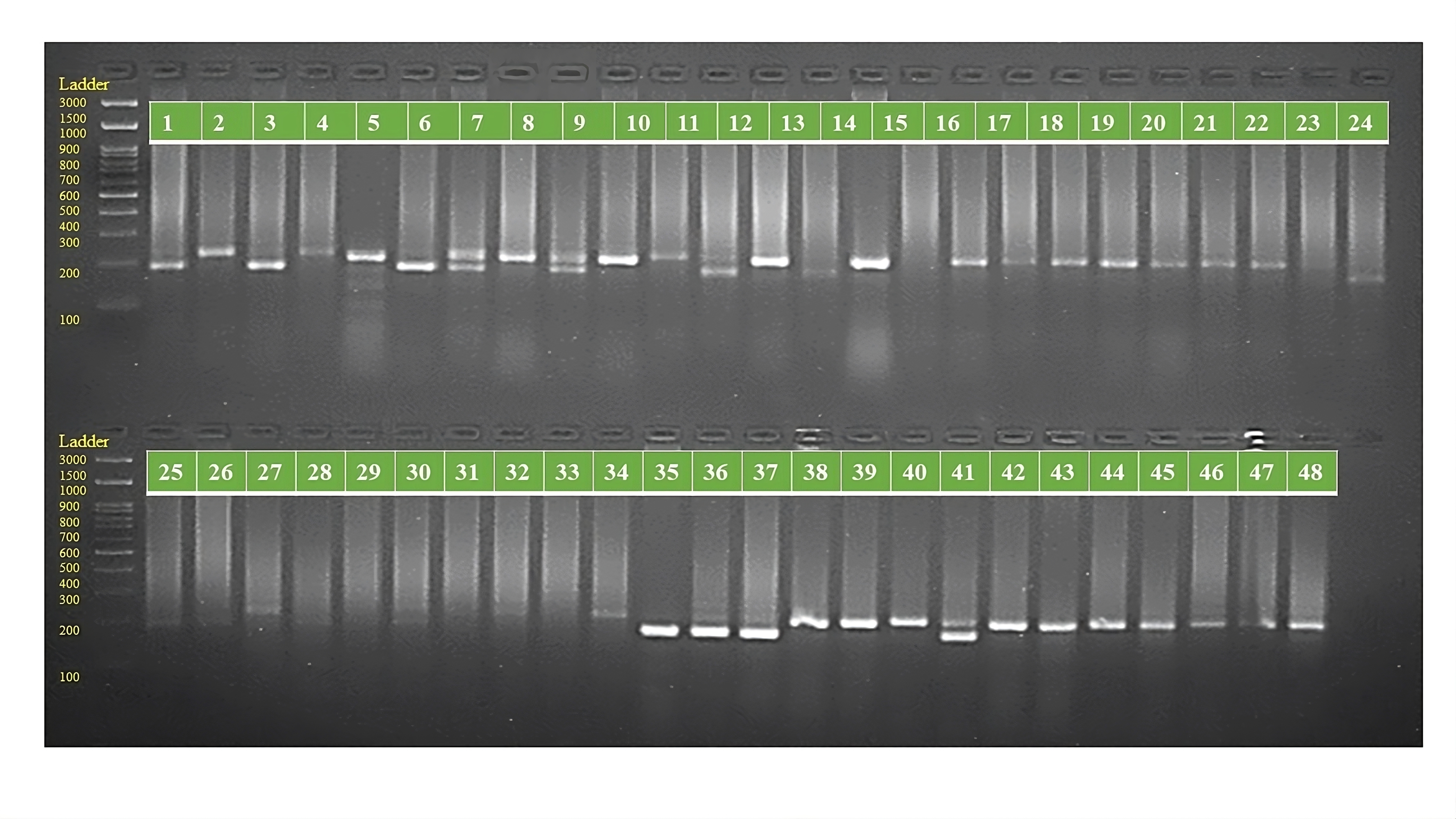

Supplement: Figure S6 — The genotypes are follows as: 1. ARM 248B, 2. COSF 6B ©, 3. GMU 1181, 4. RHA 102, 5. CMS 1103B, 6. GMU 780, 7. CMS 335B, 8. RCR 72, 9. CMSNDCMS2B, 10. GMU 344, 11. CSFI 99 ©, 12. RHA 272-1, 13. GMU 336, 14. RHA GPR 58, 15. CMS 911B, 16. GMU 477, 17. GMU 450, 18. PM 95, 19. GMU 428, 20. GMU 411, 21. GMU 741, 22. CMS 108B, 23. COSFV ©5, 24. GMU 734, 25. ARM 240B, 26. CMS 597B, 27. HOCL 6R, 28. RHA GPR 110, 29. IB 80, 30. CMS 107B, 31. PM 36, 32. IL 77, 33. RHA95-C-10, 34. IL84, 35. RHA 278, 36. RHA 273, 37. RHA 857, 38. GMU 755, 39. PM 53, 40. GP6 912, 41. CMS 135B, 42. REC 431, 43. GMU 325, 44. GP6 1089, 45. RHA 378, 46. PM 65, 47. RHA GMU 755 and 48. COSF 13B. [file peerj-12-18205-s015.jpg]

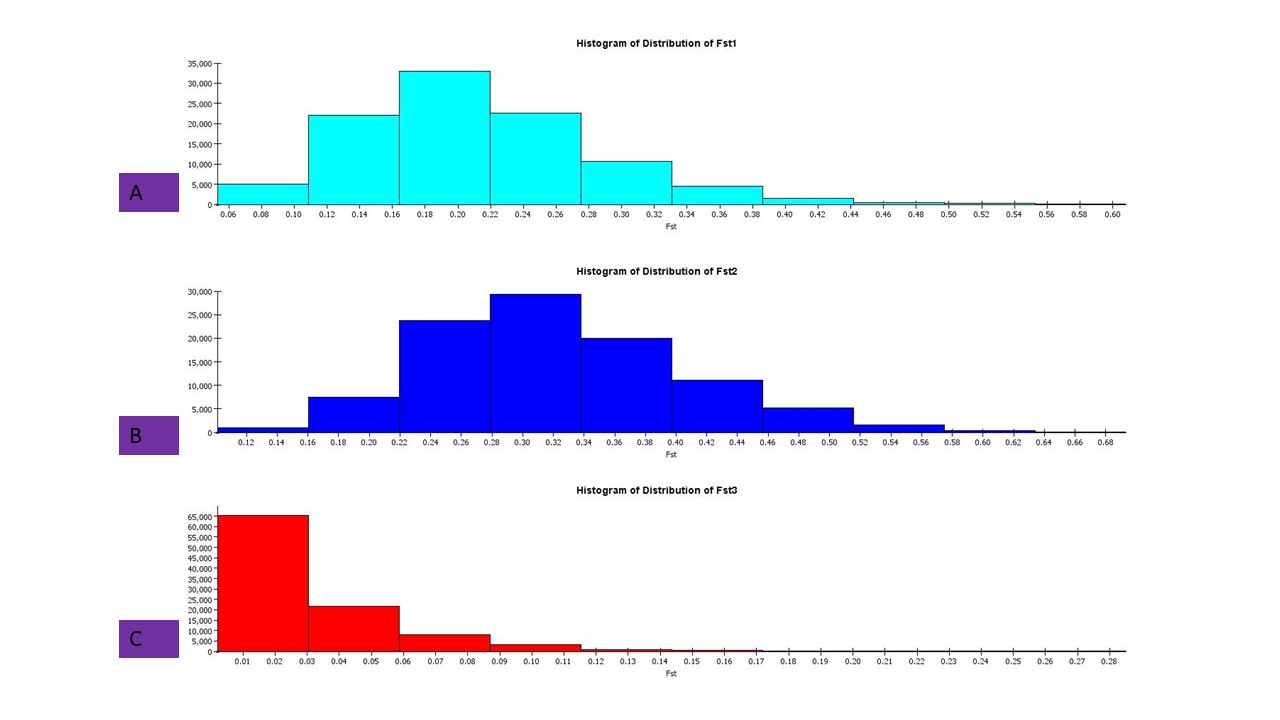

Supplement: Figure S7 — (A) Histogram distribution of Fst1, (B) Histogram distribution of Fst2 and (C) Histogram distribution of Fst3 [file peerj-12-18205-s016.jpg]

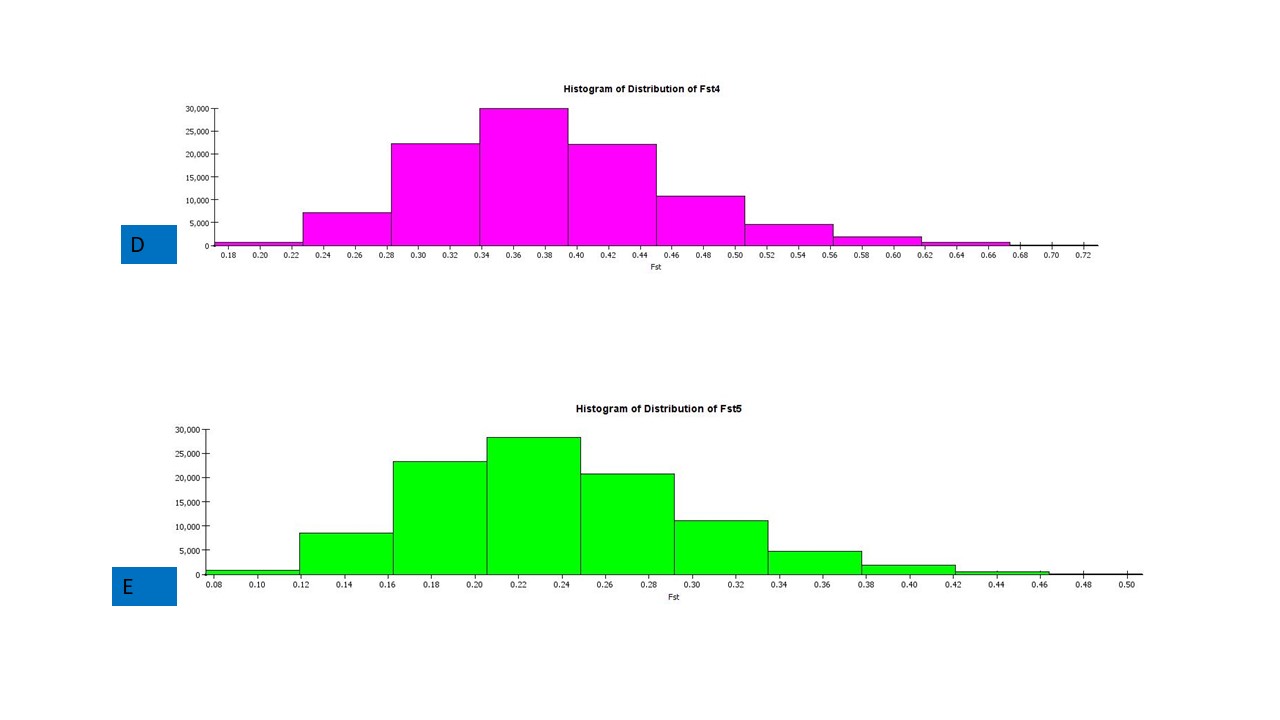

Supplement: Figure S8 — (D) Histogram distribution of Fst4 and (E) Histogram distribution of Fst5 [file peerj-12-18205-s017.jpg]
